# Postloading insulinemia is independently associated with arterial stiffness in young Japanese persons

**DOI:** 10.1038/s41440-021-00749-4

**Published:** 2021-09-13

**Authors:** Norimitsu Murai, Naoko Saito, Sayuri Nii, Yuto Nishikawa, Asami Suzuki, Eriko Kodama, Tatsuya Iida, Kentaro Mikura, Hideyuki Imai, Mai Hashizume, Yasuyoshi Kigawa, Rie Tadokoro, Chiho Sugisawa, Kei Endo, Toru Iizaka, Fumiko Otsuka, Shun Ishibashi, Shoichiro Nagasaka

**Affiliations:** 1 Division of Diabetes, Metabolism and Endocrinology, Showa University Fujigaoka Hospital, Yokohama, Kanagawa Japan; 2 Division of Endocrinology and Metabolism, Department of Medicine, Jichi Medical University, Tochigi, Japan

**Keywords:** Arterial stiffness, Insulin, Mean blood pressure, Oral glucose tolerance test, Proinsulin

## Abstract

Associations of arterial stiffness with glucose, insulin, and proinsulin dynamics during the oral glucose tolerance test (OGTT) remain under debate. The aim of this study was to investigate whether plasma glucose (PG), insulin, and proinsulin (Pro) contribute to arterial stiffness, measured by pulse wave velocity (PWV), in young Japanese persons. PG, immunoreactive insulin (IRI), and Pro levels were determined in 1193 young Japanese subjects (<40 years of age) with normal glucose tolerance or nondiabetic hyperglycemia before and at 30, 60, and 120 min during a 75-g OGTT. Participants were divided into two groups according to the median PWV. Background factors, PG, IRI, and Pro levels during the OGTT, and insulin sensitivity (SI) indices in each group were compared. Several multiple regression analysis models were used to evaluate factors contributing to PWV. All IRI and Pro levels before and after glucose loading and the area under the curve (AUC) values for IRI and Pro increased with higher PWV. 1/HOMA-IR and ISI-Matsuda as measures of SI decreased with higher PWV. The IRI AUC and Pro level before glucose loading (Pro0) were independently associated with PWV, in addition to male sex, heart rate, and mean blood pressure. The IRI AUC had a stronger relationship with PWV than Pro0. The IRI AUC had an independent relationship with PWV, whereas both SI indices did not. Postloading insulinemia, but not reduced SI, was independently associated with arterial stiffness in young Japanese persons.

## Introduction

Hyperglycemia, such as that seen in nondiabetic hyperglycemia (NDH) and diabetes mellitus (DM), is a well-known risk factor for cerebrocardiovascular diseases [1–3]. Evaluation of cerebrocardiovascular complications is important for predicting patient outcomes. Because these complications develop as a result of vascular arteriosclerotic changes, several methods have been developed to evaluate these arteriosclerotic changes. One such method is measurements of pulse wave velocity (PWV). PWV has been widely used to assess arteriosclerotic changes representing cerebrocardiovascular disease risk [4, 5].

Glucose intolerance, insulin resistance, or hyperinsulinemia during the oral glucose tolerance test (OGTT) is reported to be associated with the progression of arteriosclerosis in subjects with varying degrees of glucose intolerance [6–10]. Hyperproinsulinemia is also associated with arterial stiffness or coronary arteriosclerosis. Many cohort studies have used fasting insulin or proinsulin (Pro) levels to evaluate associations, finding a correlation between higher levels and events caused by arteriosclerotic diseases [11–16]. OGTT 1-h glucose levels are also associated with arterial stiffness [17–19], and associations are reported between 1-h OGTT glucose levels and the risk of diabetes onset, cardiovascular risk factors, and death [20–23]. These studies, however, failed to sufficiently evaluate postloading insulin or Pro levels. The association between 1-h OGTT glucose levels and arterial stiffness could be mediated by hyperinsulinemia or hyperproinsulinemia. The above studies failed to fully show whether postloading glucose, previously cited measures such as insulin or Pro levels, or insulin resistance is most relevant to arterial stiffness.

A comprehensive comparison of the associations of glucose, insulin, and Pro levels with arteriosclerosis showed hyperinsulinemia and hyperproinsulinemia on the OGTT to be related to coronary arteriosclerosis [24]. Another study, however, found that adjusting for body mass index (BMI) eliminated the association between hyperinsulinemia and hyperproinsulinemia on the OGTT and coronary artery disease [25]. It was also reported that hyperinsulinemia and insulin resistance rather than hyperglycemia on the OGTT were related to carotid artery stiffness in middle-aged, nondiabetic, hypertensive patients [26]. However, in that study, the relative importance of hyperinsulinemia and insulin resistance was unclear, and the number of participants was small (*n* = 161). To the best of our knowledge, few studies have assessed whether glucose, insulin, or Pro dynamics during the OGTT are associated with arterial stiffness. Therefore, the present study was undertaken to clarify whether glycemia, insulinemia, proinsulinemia, and/or insulin resistance is most closely associated with arterial stiffness. The study also considered the contribution of background factors such as age, sex, obesity, lipid profiles, blood pressure, and heart rate, as well as adiponectin and high-sensitivity C-reactive protein (hsCRP) levels as humoral factors, to a possible relationship with arterial stiffness [27–32]. This large-scale study with a sample size exceeding 1000 included primarily young, nonobese participants with normal glucose tolerance (NGT) and uniform background factors to minimize confounding factors.

## Materials and Methods

### Diagnosis of glucose tolerance

The criteria of the Japan Diabetes Society define NGT as fasting plasma glucose <110 mg/dL and 120-min value <140 mg/dL, impaired glucose tolerance (IGT) as fasting plasma glucose <110 mg/dL and 120-min value ≥140 mg/dL to <200 mg/dL, impaired fasting glucose (IFG) as fasting plasma glucose ≥110 mg/dL to <126 mg/dL and 120-min value <140 mg/dL, and DM as fasting plasma glucose ≥126 mg/dL and/or 120-min value ≥200 mg/dL during a 75-g OGTT [33]. NDH included IGT and/or IFG in this criteria.

### Participants

Approximately 1400 medical students of Jichi Medical University underwent a 75-g OGTT from December 2 002 to April 2 015 and of those, 1193 who were younger than 40 years of age and had NGT (*n* = 1158) or NDH (*n* = 35) were included. When participants were divided into tertiles according to the recruitment period, there were no differences in age, sex, or BMI in the tertiles (data not shown). The exclusion criteria were as follows: participants aged ≥40 years, those who showed DM on the OGTT, and those who lacked PWV measurements. None of the participants had taken antihypertensive or glucose- and lipid-lowering agents. The present study was approved by the Ethics Committee of Jichi Medical University (EKI 09–45). The participants were fully informed of the purpose of the study and provided written consent to participate.

### Measurements and calculation of indices

Plasma glucose was determined using a glucose oxidase assay, and insulin was determined using an immunoradiometric assay for immunoreactive insulin (IRI) (Insulin RIA Beads II; Yamasa, Tokyo, Japan), as described previously [34]. The manufacturer claims that there is little cross-reactivity with Pro in the immunoradiometric assay for IRI. Pro was determined as intact with the Intact-Proinsulin Assay (MLT Research, Ltd., Cardiff, UK), a chemiluminescent immunoassay procedure, as described previously **[**35]. Interassay and intraassay variabilities for insulin and intact Pro were under 5% and 10%, respectively. Samples for insulin and intact Pro analyses were frozen until immunoassays, which were performed at approximately 6-month intervals.

On the 75-g OGTT, plasma glucose, IRI, and Pro levels were measured under fasting conditions (preloading) and at 30, 60, and 120 min after glucose loading, and they are abbreviated as PG0, PG30, PG60, and PG120 (plasma glucose), IRI0, IRI30, IRI60, and IRI120 (IRI), and Pro0, Pro30, Pro60, and Pro120 (proinsulin), respectively. The area under the curve (AUC) during the OGTT was calculated for PG (PG AUC), IRI (IRI AUC), and Pro (Pro AUC). The trapezoidal rule was used to calculate the AUC. The units of PG, IRI, Pro, PG AUC, IRI AUC, and Pro AUC were mg/dL, μU/mL, pmol/L, mg·hours (h)/dL, μU·h/mL, and pmol·h/L, respectively. PG AUC, IRI AUC, and Pro AUC were employed as representatives of PG, IRI, and Pro dynamics during the OGTT.

The following measures of insulin sensitivity (SI) using fasting and/or 2-h OGTT PG and IRI levels were employed in this study to avoid multicollinearity with the IRI AUC. ISI-Matsuda, a measure of systemic SI, was calculated as follows: ISI-Matsuda = 10 000/[sqrt(PG0 ∙ PG120 ∙ IRI0 ∙ IRI120)] [36, 37]. In addition, 1/homeostasis model assessment of insulin resistance (HOMA-IR) was used primarily as a measure of hepatic SI. HOMA-IR was calculated as [PG0·IRI0]/405 [38]. For ISI-Matsuda and HOMA-IR, the units of PG and IRI were mg/dL and μU/mL, respectively.

Total cholesterol (T-C), triglyceride (TG), high-density lipoprotein cholesterol (HDL), adiponectin, and hsCRP levels of the participants were determined using serum collected under fasting conditions. Low-density lipoprotein cholesterol (LDL) concentration was calculated using the Friedewald formula [39]. Total adiponectin and hsCRP concentrations were measured using an ELISA kit (Otsuka Pharmaceutical, Tokyo, Japan) and an ultrahigh-sensitivity latex turbidimetric immunoassay (Behring Nephelometry, Tokyo, Japan), respectively.

BMI was calculated as the weight in kilograms divided by the height in meters squared. Waist circumference (WC) was measured at the umbilical level while standing.

Brachial-ankle PWV (baPWV) was measured with an oscillometric device (FORM, Fukuda Denshi, Tokyo, Japan) after the subjects had been at rest for 5 min in a supine position. Briefly, pressure waveforms of the brachial and tibial arteries were obtained using occluding monitoring cuffs placed around the upper arms and lower legs. Times taken for pulse waves to travel from the lower legs to the upper arms were recorded; distances between sampling points were calculated automatically from subject heights. baPWV was defined as the mean of left and right baPWV. Heart rate (HR) was determined, and systolic and diastolic blood pressures (SBP and DBP) were calculated as the mean pressures obtained from the left and right arms, both measured with the same device. Mean blood pressure (MBP) was calculated as DBP + (SBP − DBP)/3.

### Statistical analysis

JMP version 5.1 (SAS Institute Inc., Cary, NC, USA) was used to conduct the statistical analyses. Most variables were not normally distributed. Values are shown as medians (25th percentile, 75th percentile). The Wilcoxon signed-rank test or the chi-squared test was used to test for differences between the two groups.

First, the participants were divided into two groups according to their median baPWV. Background factors in the two groups were compared. PG, IRI, Pro, PG AUC, IRI AUC, and Pro AUC, which were considered to constitute glucose-insulin-proinsulin profiles, were compared in the groups. Then, measures of SI, 1/HOMA-IR, and ISI-Matsuda were compared.

Multiple regression analysis was used to calculate regressions for baPWV. When conducting multiple regression analysis, the background factors that were significantly different between the high- and low-baPWV groups (i.e., age, number of males, BMI, WC, HR, SBP, DBP, MBP, HDL, TG, and adiponectin levels) were used as explanatory variables. At this time, pairs of variables with a variance inflation factor >2 (SBP, DBP and MBP; WC and BMI) were considered to have multicollinearity, and variables with a lower coefficient of correlation with baPWV (SBP and DBP, and WC) were excluded. Included in the multiple regression analyses in addition to the background factors were the glucose-insulin-proinsulin profiles of fasting indices (PG0, IRI0, and Pro0) or AUC indices (PG AUC, IRI AUC, and Pro AUC), or both, or Pro0 and IRI AUC. To determine whether SI or insulinemia contributes to baPWV, another multiple regression analysis was conducted that included, in addition to the background factors, one of the SI indices (1/HOMA-IR and ISI-Matsuda) or IRI AUC, or all of these.

In all statistical tests, a *P* value < 0.05 was taken to indicate significance.

## Results

### Participants’ background factors according to baPWV

The participants’ background factors overall and according to baPWV are shown in Table 1. The median baPWV (25th percentile, 75th percentile) was 1122 (1025, 1220) cm/s (baPWV < 1 122 cm/s, *n* = 595; baPWV ≥ 1 122 cm/s *n* = 598). The participants were young, and no significant differences in LDL, T-C, frequency of NDH, or hsCRP levels were seen between baPWV groups. Age, number of males, BMI, WC, HR, SBP, DBP, MBP, and TG levels increased with higher baPWV, but HDL and adiponectin levels decreased with higher baPWV.

**Table 1:** Background factors in overall participants and comparison of those according to baPWV

	Overall *n* = 1193	baPWV low <1122 (cm/seconds) *n* = 595	baPWV high ≥1122 (cm/seconds) *n* = 598	*P* value
Age (year)	22 (22, 23)	23 (22, 23)	23 (22, 23)	<0.01
Male/Female (number)	Male 934/Female 259	Male 415/Female 180	Male 519/Female 79	<0.0001
BMI (kg/m^2^)	21 (20, 23)	21 (20, 23)	22 (20, 24)	<0.0001
WC (cm)	75 (71, 79)	74 (70, 79)	76 (72, 81)	<0.0001
HR (/minutes)	63 (57, 70)	61 (55, 68)	65 (58, 73)	<0.0001
SBP (mmHg)	118 (111, 126)	114 (107, 120)	123 (116, 130)	<0.0001
DBP (mmHg)	66 (62, 72)	63 (60, 68)	70 (65, 75)	<0.0001
MBP (mmHg)	84 (79, 90)	80 (75, 85)	88 (83, 93)	<0.0001
HDL (mg/dL)	61 (53, 69)	62 (55, 71)	59 (52, 67)	<0.0001
TG (mg/dL)	60 (45, 80)	56 (43, 73)	64 (49, 89)	<0.0001
LDL (mg/dL)	91 (76, 106)	90 (76, 105)	92 (76, 108)	0.33
T-C (mg/dL)	166 (149, 184)	166 (148, 184)	167 (151, 184)	0.54
NDH/NGT	35/1158	18/577	17/581	0.85
Adiponectin (μg/mL)	6.9 (4.9, 9.2)	7.5 (5.3, 9.7)	6.3 (4.5, 8.6)	<0.0001
hsCRP (ng/mL)	212 (101, 478)	194 (97, 428)	229 (107, 494)	0.092

Data are shown as median (25th percentile, 75th percentile) or number

The participants were divided into two groups according to their median baPWV. The Wilcoxon signed-rank test or the chi-squared test was used to test for differences between the two groups (*P* value, baPWV low vs. baPWV high)

*baPWV* brachial-ankle pulse wave velocity, *BMI* body mass index, *WC* waist circumference, *HR* heart rate, *SBP* systolic blood pressure, *DBP* diastolic blood pressure, *MBP* mean blood pressure, *HDL* high-density lipoprotein cholesterol, *TG* triglycerides, *LDL* low-density lipoprotein cholesterol, *T-C* total cholesterol, *NGT* normal glucose tolerance, *NDH* nondiabetic hyperglycemia, *hsCRP* high-sensitivity C-reactive protein

### Glucose-insulin-proinsulin profiles and SI according to baPWV

**Fig. 1:**
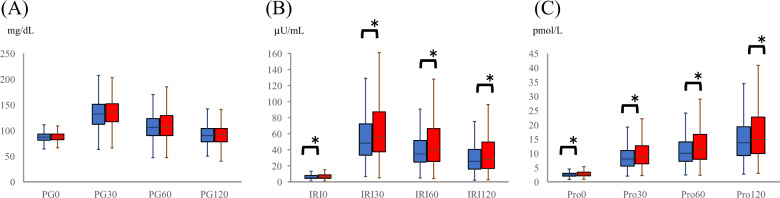
Glucose, insulin, and proinsulin levels during the OGTT according to baPWV. Box-and-whisker plot graphs for glucose (**A**), insulin (**B**), and proinsulin (**C**) levels according to baPWV are shown. OGTT oral glucose tolerance test, baPWV brachial-ankle pulse wave velocity, PG plasma glucose, IRI immunoreactive insulin, Pro proinsulin. Blue boxes are baPWV low, and red boxes are baPWV high. *P* values for the variables were determined by the Wilcoxon signed-rank test. *P < 0.05

**Fig. 2:**
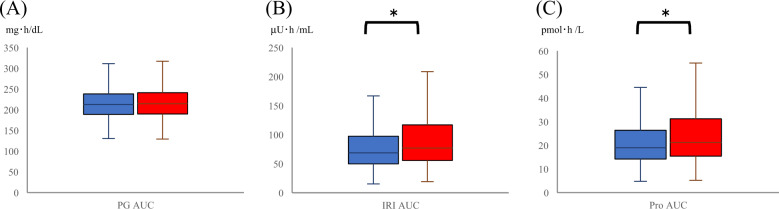
PG AUC, IRI AUC, and Pro AUC during the OGTT according to baPWV. Box-and-whisker plot graphs for PG AUC (**A**), IRI AUC (**B**), and Pro AUC (**C**) according to baPWV are shown. PG plasma glucose, AUC area under the curve, IRI immunoreactive insulin, Pro proinsulin, OGTT oral glucose tolerance test, baPWV brachial-ankle pulse wave velocity. Blue boxes are baPWV low, and red boxes are baPWV high. *P* values for the variables were determined by the Wilcoxon signed-rank test. *P < 0.05

**Fig. 3:**
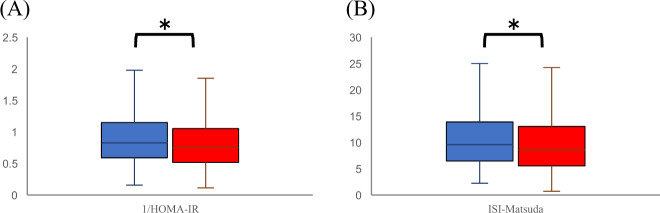
1/HOMA-IR and ISI-Matsuda according to baPWV. Box-and-whisker plot graphs for 1/HOMA-IR (**A**) and ISI-Matsuda (**B**) according to baPWV are shown. HOMA-IR homeostasis model assessment of insulin resistance, ISI-Matsuda Matsuda index, baPWV brachial-ankle pulse wave velocity. Blue boxes are baPWV low, and red boxes are baPWV high. *P* values for the variables were determined by the Wilcoxon signed-rank test. *P < 0.05

PG, IRI, and Pro levels during the OGTT (Fig. 1), PG AUC, IRI AUC, and Pro AUC (Fig. 2), and 1/HOMA-IR and ISI-Matsuda (Fig. 3) according to baPWV are shown. PG levels during the OGTT (Fig. 1) and PG AUC (Fig. 2) were not significantly different between baPWV groups. All IRI and Pro levels during the OGTT (Fig. 1), IRI AUC, and Pro AUC (Fig. 2) increased with higher baPWV. 1/HOMA-IR and ISI-Matsuda decreased with higher baPWV (Fig. 3).

### Multiple regression analysis of background factors and glucose-insulin-proinsulin profiles for baPWV

The results of multiple regression analyses of background factors and glucose-insulin-proinsulin profiles for baPWV are shown in Table 2. All models showed baPWV to be positively correlated with male sex, HR, and MBP and negatively correlated with BMI. HDL, TG, and adiponectin levels were not significantly correlated. In addition to the background factors, Model 1 included fasting indices, and Model 2 included AUC indices. The analysis showed positive correlations of baPWV with Pro0 in Model 1. The analysis also showed positive correlations of baPWV with IRI AUC in Model 2. Model 3 included all fasting and AUC indices; the analysis showed positive correlations of baPWV with IRI AUC. Model 4 included Pro0, which was shown to have a significant correlation in Model 1, and IRI AUC, which was shown to have a significant correlation in Model 2. The analysis showed positive correlations of baPWV with IRI AUC.

**Table 2:** Multivariate regression analysis of background factors and glucose-insulin-proinsulin profiles for baPWV

	Model 1		Model 2		Model 3		Model 4	
	*t* value	*p* value	*t* value	*p* value	*t* value	*p* value	*t* value	*p* value
Age	0.86	0.39	0.86	0.39	0.88	0.38	0.83	0.41
Male proportion	3.05	<0.01	3.18	<0.01	3.19	<0.01	3.20	<0.01
BMI	−3.82	<0.001	−4.24	<0.0001	−4.13	<0.0001	−4.38	<0.0001
HR	4.10	<0.0001	3.80	<0.001	3.86	<0.001	3.88	<0.001
MBP	15.81	<0.0001	16.08	<0.0001	16.00	<0.0001	15.97	<0.0001
HDL	−1.12	0.26	−1.14	0.25	−1.00	0.32	−1.05	0.29
TG	1.05	0.29	0.83	0.41	0.80	0.42	0.73	0.46
Adiponectin	−0.78	0.44	−0.60	0.55	−0.71	0.48	−0.63	0.53
PG0	−1.46	0.14			−1.48	0.14		
IRI0	−0.78	0.44			−0.77	0.44		
Pro0	2.08	<0.05			0.80	0.42	0.82	0.41
PG AUC			0.020	0.99	0.50	0.61		
IRI AUC			2.00	<0.05	2.02	<0.05	2.54	<0.05
Pro AUC			0.96	0.34	0.050	0.96		

*baPWV* brachial-ankle pulse wave velocity, *BMI* body mass index, *HR* heart rate, *MBP* mean blood pressure, *HDL* high- density lipoprotein cholesterol, *TG* triglycerides, *PG* plasma glucose, *IRI* immunoreactive insulin, *Pro* proinsulin, *AUC* area under the curve

Model 1; background factors and PG0, IRI0, and Pro0

Model 2; background factors and PG AUC, IRI AUC, and Pro AUC

Model 3; background factors and PG0, IRI0, Pro0, PG AUC, IRI AUC, and Pro AUC

Model 4; background factors and Pro0 and IRI AUC

### Multiple regression analyses of background factors, SI indices, and IRI AUC for baPWV

The results of multiple regression analysis of background factors, SI indices, and IRI AUC for baPWV are shown in Table 3. All models showed baPWV to be positively correlated with male sex, HR, and MBP and negatively correlated with BMI. The analysis showed no correlations of SI indices (1/HOMA-IR and ISI-Matsuda) with baPWV in Models 1 and 2. The analysis showed positive correlations of IRI AUC with baPWV in Models 3 and 4.

**Table 3:** Multivariate regression analysis of background factors, indices of SI, and IRI AUC for baPWV

	Model 1		Model 2		Model 3		Model 4	
	*t* value	*p* value	*t* value	*p* value	*t* value	*p* value	*t* value	*p* value
Age	0.85	0.40	0.88	0.38	0.87	0.38	0.83	0.41
Male proportion	2.93	<0.01	3.05	<0.01	3.20	<0.01	3.10	<0.01
BMI	−3.76	<0.001	−3.89	<0.001	−4.32	<0.0001	−4.20	<0.0001
HR	4.02	<0.0001	3.84	<0.001	3.87	<0.001	3.92	<0.0001
MBP	16.08	<0.0001	16.02	<0.0001	16.14	<0.0001	16.15	<0.0001
HDL	−1.22	0.22	−1.07	0.29	−1.06	0.29	−1.09	0.27
TG	1.41	0.16	1.31	0.19	0.90	0.37	0.96	0.34
Adiponectin	−0.74	0.46	−0.76	0.45	−0.66	0.51	−0.65	0.52
1/HOMA-IR	0.040	0.97					0.81	0.42
ISI-Matsuda			−1.06	0.29			0.020	0.99
IRI AUC					2.92	<0.01	2.87	<0.01

*SI* insulin sensitivity, *IRI AUC* the area under the curve of immunoreactive insulin, *baPWV* brachial-ankle pulse wave velocity, *BMI* body mass index, *HR* heart rate, *MBP* mean blood pressure, *HDL* high-density lipoprotein cholesterol, *TG* triglycerides, *HOMA-IR* homeostasis model assessment of insulin resistance, *ISI-Matsuda* Matsuda index

Model 1; background factors and 1/HOMA-IR

Model 2; background factors and ISI-Matsuda

Model 3; background factors and IRI AUC

Model 4; background factors and 1/HOMA-IR, ISI-Matsuda and IRI AUC

## Discussion

The comparative relevance of glycemia, insulinemia, and proinsulinemia during the OGTT to PWV is unclear. The present study, which included primarily young individuals with NGT, showed IRI AUC and Pro0 to be independently correlated with baPWV in addition to the background factors of male sex, HR, and MBP, with no contribution by postloading glycemia. Multiple regression analysis that included both IRI AUC and Pro0 eliminated a significant correlation between Pro0 and baPWV, showing IRI AUC to be independently correlated with baPWV. SI indices (1/HOMA-IR and ISI-Matsuda) were not significantly correlated with baPWV, and this analysis again showed IRI AUC to be independently correlated with baPWV. The humoral factors adiponectin and hsCRP did not contribute to baPWV. Postloading insulinemia was independently associated with arterial stiffness in young Japanese persons. Insulin hypersecretion, rather than insulin resistance, seemed to be related to arterial stiffness in this population.

First, the association of baPWV with background factors of the participants was investigated. BMI was higher in the high-baPWV group than in the low-baPWV group (Table 1). Multivariate analysis showed an inverse correlation between BMI and baPWV (Tables 2 and 3), indicating a lack of association of BMI with baPWV. Male sex, HR, and MBP increased independently with higher baPWV. hsCRP was not significantly different between the low- and high-baPWV groups. Multivariate analysis did not show an independent correlation of lipid profiles and adiponectin with baPWV. The associations of male sex, HR, and blood pressure with PWV were consistent with previous studies [27–29]. However, the present study did not identify associations of BMI, lipid profiles, or hsCRP with baPWV, as seen in another study [30]. This disparity might be explained by the subject’s characteristics in the present study, the mostly young, nonobese population, [30]. Studies in elderly patients who were hyperglycemic, hypertensive, and had metabolic syndrome showed a negative correlation between adiponectin and PWV [31, 32]. We are unaware of any studies that have examined the association between adiponectin and baPWV in a population of individuals primarily with NGT, and in the uniform population of the present study, adiponectin was not independently correlated with baPWV.

The next analysis considered the association of background factors and glucose-insulin-proinsulin profiles with baPWV. Pre- and postloading glucose and PG AUC did not differ by baPWV. Previous studies, however, found a correlation between PG60 and arterial stiffness [17–19]. A PG60 of 155 mg/dL has been proposed as a cutoff value for diabetes onset and death regardless of glucose tolerance status [23], and in a study of patients with confirmed hypertension (mean age ~40 years, NGT or IGT), patients with PG60 ≥ 155 mg/dL had a significantly higher PWV than patients with PG60 < 155 mg/dL [18]. The high blood pressure of the participants, however, may have substantially affected PWV in this report. Another study in individuals with normal blood pressure and NGT identified an association between PG60 and baPWV, but it did not investigate the effects of age or sex [17]. However, another study in nonhypertensive individuals with NGT showed a significant correlation between PG60 and baPWV in men ≥45 years of age and no such correlation in men <45 years of age or women [19]. Neither study considered insulinemia in detail [17–19]. Although many of the participants in the present study were men, only 8.0% had PG60 ≥ 155 mg/dL, which is much lower than the 34.4% of a previous study [18]. The participants in the present study were young and mostly normoglycemic and normotensive. In this young population, baPWV was not associated with postloading glucose.

Pre- and postloading IRI, and Pro levels were higher in the high-baPWV group. IRI AUC and Pro0, as well as the background factors of male sex, HR, and MBP, were independently and positively correlated with baPWV. These findings agree with the correlation between high postloading IRI and high PWV found in multiple studies [6, 18, 24] and with a postulated independent correlation between insulin secretion and PWV with minimal effects of elevated glucose on PWV in a study using the glucose clamp method [40]. Pro0 was positively correlated with IRI AUC in the present study (Spearman’s test; correlation coefficient 0.29, *P* < 0.0001). Multiple regression analysis of the correlation of Pro 0 and IRI AUC with baPWV showed no significant contribution of Pro 0, demonstrating that IRI AUC contributed independently to baPWV. In studies that investigated the correlation of fasting Pro with arterial stiffness or coronary arteriosclerosis [11–16], postloading hyperinsulinemia may have mediated the correlations identified. The results of the present study suggest that postloading insulinemia rather than Pro may contribute to arterial stiffness.

Although the cause-and-effect relationship is unclear, hyperinsulinemia and insulin resistance are observed concurrently [41]. Hyperinsulinemia causes arteriosclerosis via pathogenic cellular mechanisms, and insulin resistance does so via dyslipidemia and/or elevated inflammatory markers [42]. Whether hyperinsulinemia or insulin resistance contributes more to arterial stiffness is an unresolved question [42]. In the present study, the SI indices 1/HOMA-IR and ISI-Matsuda were compared to the hyperinsulinemia index IRI AUC in terms of associations with baPWV. Multivariate analysis showed that neither of the SI indices were correlated, but the IRI AUC was positively correlated with baPWV. Studies with relatively uniform populations (NGT, nonhypertensive, children) have found insulin secretion and insulin resistance to be related to arterial stiffness [6, 8, 9]. The participants in these studies had a relatively high degree of obesity or, in the case of the child participants, a low degree of obesity but were a few in number. The results of the present study, with its population of primarily young, nonobese individuals with NGT, suggest that postloading insulinemia rather than insulin resistance may contribute to arterial stiffness.

Hyperinsulinemia could accelerate the arteriosclerotic process by multiple mechanisms, including vascular smooth muscle cell growth and proliferation, activation of genes involved in inflammation, increased collagen synthesis, and enhanced LDL cholesterol transport into arterial smooth muscle cells [42]. Hyperinsulinemia is also induced by impaired hepatic insulin clearance in diseases such as fatty liver [43], which is known to be associated with arteriosclerosis [44]. In the present study, liver function tests were not performed, but almost all participants were not obese, and their WC was normal. Given that, it seems unlikely that the association of hyperinsulinemia with baPWV was due to fatty liver and impaired hepatic insulin clearance. When baPWV values were divided into quartiles, IRI AUC in the quartiles increased incrementally [Q1 (baPWV < 1025, *n* = 298): 67 (47, 96), Q2 (1025 ≤ baPWV < 1122, *n* = 297): 70 (51, 98), Q3 (1122 ≤ baPWV < 1219, *n* = 298): 73 (52, 110), Q4 (1219 ≤ baPWV, *n* = 300): 81 (58, 130), respectively]; the relationship between baPWV and postloading insulinemia seemed to be linear.

Several limitations must be noted. Due to the cross-sectional nature of the study, a direct causal relationship between postloading insulinemia and baPWV was not proven. The fact that the participants were young Japanese individuals who were mostly nonobese and had low baPWV means that the results may not be generalizable to people of other ethnicities, patients with metabolic syndrome or glucose intolerance, and/or patients with borderline and abnormal baPWV [45]. However, the strength of the present study is that background, insulin, and Pro dynamics during the OGTT, and baPWV were examined using a large sample of participants with common characteristics (mainly NGT and young). The investigators had to use OGTT-based SI indices, since the glucose clamp method or minimal model analysis would not have been feasible in this large sample size. The SI indices used, however, are closely correlated with SI derived from the glucose clamp method [36, 37]. Unfortunately, we did not have exact data regarding the smoking status and exercise habits of the participants. However, we recognize that there were very few smokers among the participants, and we believe that the effects of smoking on baPWV would be modest. The effects of exercise habits on baPWV and insulinemia require further investigation. In addition, we did not have data regarding the menstrual cycle in the female participants, but the number of females was not small (*n* = 259). Therefore, the contribution of different menstrual cycles to insulinemia was likely counterbalanced. Some of the participants in the present study could possibly develop glucose intolerance and/or arteriosclerotic diseases in the future, and a comparative study between this study and disease onset may be of potential interest.

In conclusion, postloading insulinemia was independently associated with arterial stiffness in young Japanese persons. Insulin hypersecretion, rather than insulin resistance, seemed to be related to arterial stiffness in this population.
